# Detecting grain-scale plastic deformation events with time-resolved far-field high-energy diffraction microscopy

**DOI:** 10.1107/S1600576725007009

**Published:** 2025-09-12

**Authors:** Yuefeng Jin, Wenxi Li, Amlan Das, Katherine Shanks, Ashley Bucsek

**Affiliations:** a Department of Mechanical Engineering, University of Michigan, Ann Arbor, Michigan, USA; b Department of Materials Science and Engineering, University of Michigan, Ann Arbor, Michigan, USA; c Cornell High Energy Synchrotron Source, Ithaca, New York, USA; SLAC National Accelerator Laboratory, Menlo Park, USA

**Keywords:** X-ray characterization, far-field high-energy diffraction microscopy, plastic deformation, titanium–aluminium alloys, creep loading

## Abstract

Far-field high-energy diffraction microscopy (ff-HEDM) bridges a critical gap between microscale and macroscale plasticity by enabling three-dimensional (3D) time-resolved observations of grain-scale deformation. It can be used to measure the grain-averaged elastic strain tensor, crystallographic orientation, centroid and relative volume of each individual grain. Researchers have also proposed methods to extract information about grain-scale plastic deformation from time-resolved ff-HEDM measurements, using *e.g.* signature changes in a grain’s equivalent or resolved shear stress, orientation or diffraction peak width. However, the accuracy of these different methods is largely unexplored due to the absence of an independent ground truth, particularly for plastic deformation that occurs prior to the macroscopic yield point. In the present work, we evaluate four methods for detecting grain-scale plastic deformation events using ff-HEDM: (i) equivalent stress relaxation, (ii) resolved shear stress relaxation, (iii) orientation change and (iv) diffraction peak shape evolution. Using ff-HEDM data from room-temperature creep tests of a Ti–7Al alloy, we cross-validate these approaches. The achieved high validation rates support confidence in the identified events. Two types of stress relaxation are observed among the detected events – fast and large versus gradual and small – suggesting different deformation mechanisms. The spatiotemporal distribution of plastic events is also captured, revealing clustered activity and intergranular propagation. These findings open avenues for future studies to explore the initiation and propagation of plasticity among grains.

## Abstract 2

This study evaluates four methods for detecting grain-scale plastic deformation using far-field high-energy diffraction microscopy on a Ti–7Al alloy under room-temperature creep testing. High validation rates among these detection methods support confidence in detecting plastic events without a ground truth. Two types of events – showing sharp or gradual stress relaxation – suggest different deformation mechanisms, and spatial–temporal mapping reveals clustered activity and intergranular propagation.

## 1. Introduction

Plastic deformation in ductile materials is often modeled as a smooth continuous process that occurs uniformly in space and time. However, in polycrystalline materials, plasticity often manifests as discrete localized events on the grain scale (Weiss, 2019 ▸). These grain-scale plastic events are characterized by sudden bursts of plastic deformation, resulting in plasticity that occurs heterogeneously across the material, challenging traditional continuum-based descriptions of plasticity.

The study of grain-scale plasticity has gained considerable attention, with various experimental techniques employed to observe these phenomena across different length scales. Transmission electron microscopy (TEM) has provided atomic-scale insights into dislocation dynamics (Oh *et al.*, 2006 ▸), while techniques such as acoustic emission and micropillar compression have enabled microscale observations with time resolution, primarily in single crystals (Uchic *et al.*, 2009 ▸; Dimiduk *et al.*, 2006 ▸; Drozdenko *et al.*, 2016 ▸; Alava *et al.*, 2014 ▸; Weiss *et al.*, 2007 ▸). In polycrystalline materials, scanning-electron-microscopy-based digital image correlation combined with electron backscatter diffraction (EBSD) is commonly used to study plasticity at both inter- and intra-granular levels (Echlin *et al.*, 2016 ▸; Linne *et al.*, 2020 ▸; Stinville *et al.*, 2016 ▸). However, these techniques are limited in their ability to capture the full three-dimensional (3D) nature of the deformation across bulk polycrystalline materials.

In recent years, far-field high-energy X-ray diffraction microscopy (ff-HEDM) has emerged as a powerful tool for investigating grain-scale plastic deformation in polycrystalline materials. This nondestructive 3D *in situ* technique enables the characterization of the centroid, the relative volume, the grain-averaged crystallographic orientation and the grain-averaged elastic strain tensor for each individual grain, across many hundreds to thousands of grains simultaneously (Poulsen *et al.*, 2001 ▸; Poulsen, 2004 ▸; Bernier *et al.*, 2011 ▸; Oddershede *et al.*, 2010 ▸). By providing time-resolved data during mechanical loading, ff-HEDM can provide rich insights into the transient nature of grain-scale deformation in polycrystalline systems.

Researchers have developed different methods for extracting information on grain-scale plasticity from ff-HEDM measurements. Tang *et al.* (2015 ▸) used ff-HEDM to study the elasto-viscoplastic response of the magnesium alloy AZ31B. A softening response on the grain scale was identified in lattice strain measured by ff-HEDM, and concurrent shifts in diffraction peak intensities were observed during stress relaxation. Beaudoin *et al.* (2017 ▸) employed ff-HEDM to study plastic deformation intermittency in bulk polycrystalline materials, where relaxation in effective stress derived from ff-HEDM measurements was interpreted as an indicator of grain-scale plastic deformation. More recently, Borasi, Worsnop and co-workers showed that local plastic deformation correlates with resolved shear stress (RSS) relaxation on slip systems, which can also be quantified via ff-HEDM (Borasi & Mortensen, 2024 ▸; Worsnop *et al.*, 2022 ▸). Beyond stress relaxation, researchers have also utilized other indicators of plasticity. Lim *et al.* (2022 ▸) used ff-HEDM to study grain reorientation and stress evolution in Ti alloys during cyclic loading. Their results show that, as plastic deformation accumulates, the grain orientation will change sufficiently around specific axes to be resolved by ff-HEDM. Similarly, microstructural evolution, such as orientation changes, can influence diffraction peak shapes. Pagan and co-workers established a connection between diffraction peak shape evolution and slip activity, first for single-slip systems (Pagan & Miller, 2014 ▸) and later for multiple-slip systems (Pagan & Miller, 2016 ▸). Wang *et al.* (2021 ▸) utilized diffraction peak broadening to estimate geometrically necessary dislocation (GND) densities, enabling evaluation of Taylor hardening models at the grain scale. While Wang’s study focused on macroscopic plastic deformation, Chatterjee *et al.* (2019 ▸) observed diffraction peak intensity fluctuations associated with grain-scale plasticity events that occurred within the macroscopic elastic regime during time-resolved measurements.

Given the different methods available, a comprehensive study of best practices for characterizing grain-scale plasticity with ff-HEDM is urgently needed. Currently, it remains unclear which method is most accurate, robust or generally applicable. Unlike microscopy techniques that provide direct visual evidence of dislocation or slip band structures, ff-HEDM relies on indirect metrics such as stress relaxation, lattice reorientation and diffraction peak evolution. Furthermore, there is no equivalent technique that can be applied in parallel to ff-HEDM, resulting in a lack of independent ground truth measures. However, the different plasticity-related measurements that are extractable from ff-HEDM (*e.g.* stress relaxation, lattice reorientation and diffraction peak evolution) can be exploited for validation purposes as a ground truth substitute.

**Figure 1:**
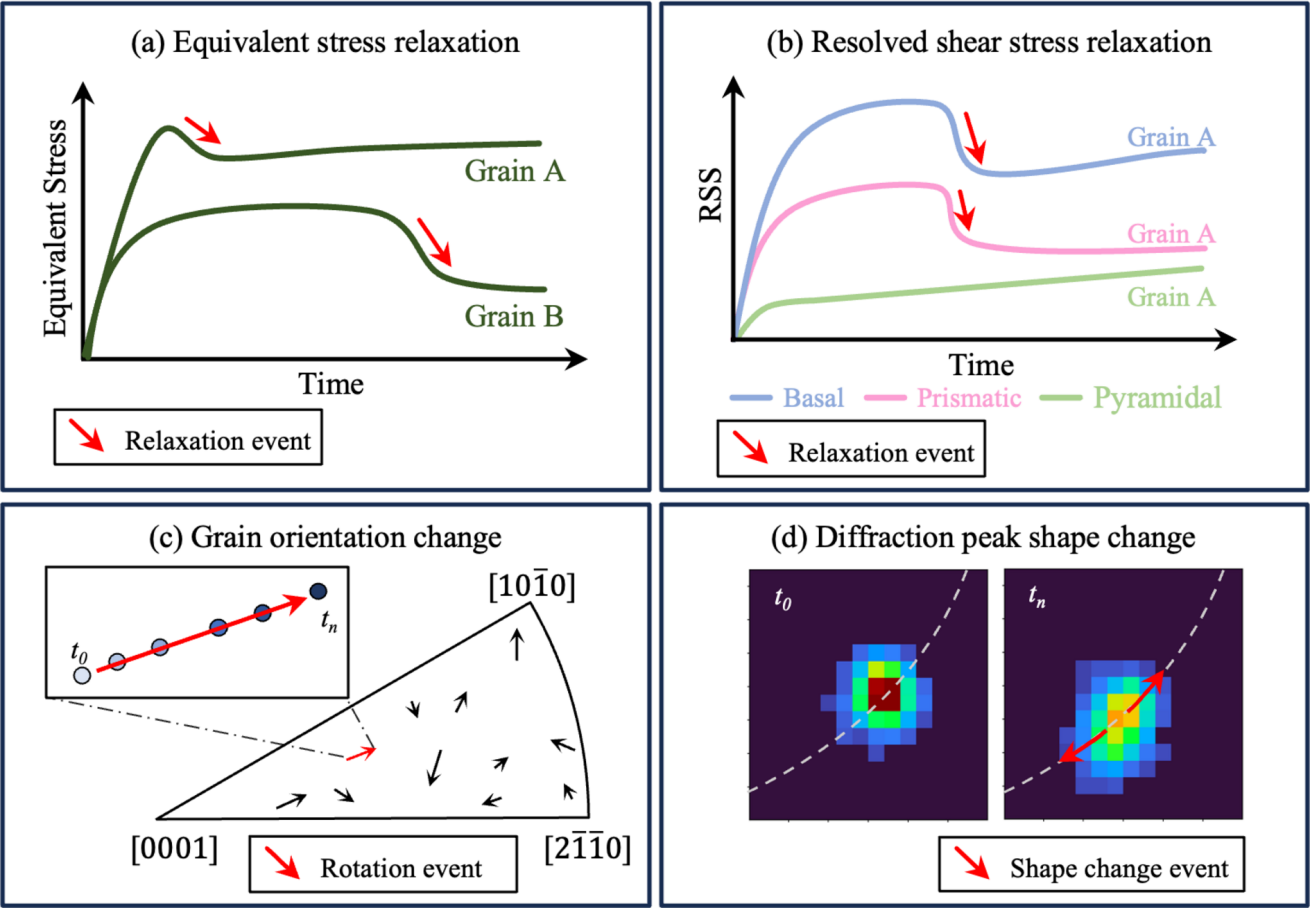
Overview of the work, showing the different methods for detecting grain-scale plastic deformation events using ff-HEDM. (*a*) Equivalent stress relaxation (Method 1), (*b*) RSS relaxation (Method 2), (*c*) orientation change (Method 3) and (*d*) diffraction peak shape change (Method 4).

In this study, we present a comprehensive evaluation of four different methodologies for detecting grain-scale plastic deformation events using ff-HEDM (Fig. 1 ▸):

Method 1: equivalent stress relaxation. Continuous decrease in the grain-averaged equivalent stress over time.

Method 2: resolved shear stress relaxation. Continuous decrease in grain-averaged RSS over time on one or more slip systems.

Method 3: orientation change. Net change in the grain-averaged orientation over loading, quantified by misorientation relative to the initial state.

Method 4: diffraction peak shape change. Accumulated broadening and intensity shift of the diffraction peak over time.

Among these, equivalent stress relaxation and RSS relaxation are used as detection methods, while orientation change and diffraction peak shape change are used for validation. This study uses ff-HEDM data collected during a room-temperature creep test on hexagonally close-packed (h.c.p.) α-phase Ti–7Al. By cross-validating between different methods, we aim to identify the most accurate and robust method to identify grain-scale plastic deformation events using ff-HEDM.

## 2. Experiment

The material used in this work is α-phase Ti–7Al with an h.c.p. crystal structure. It was manufactured by casting and extrusion, followed by annealing at 962°C for 24 h and then air cooling. This processing resulted in equiaxial grains with an average size of approximately 75 µm. Previous researchers using the same material reported that the air-cooling process promotes the short-range ordering of Ti and Al, forming nanoscale Ti_3_Al precipitates that enhance planar slip (Pagan *et al.*, 2023 ▸; Hémery *et al.*, 2020 ▸). The nominal yield strength of this material is 610 MPa (Beaudoin *et al.*, 2017 ▸).

**Figure 2:**
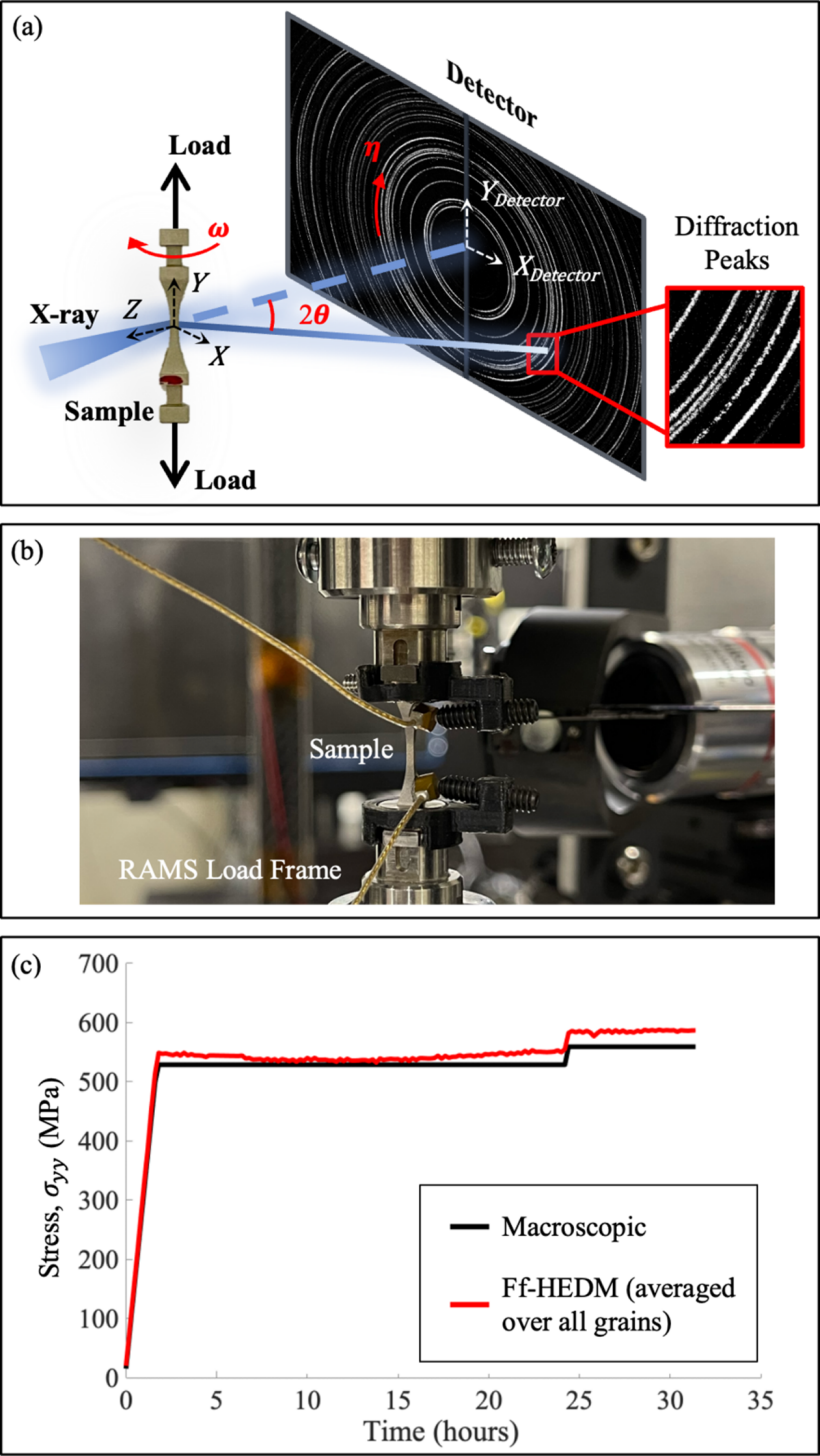
(*a*) The ff-HEDM experiment setup, where ω is the sample rotation angle, 2θ is the diffraction angle and η is the azimuthal angle. (*b*) Sample placed in the RAMS load frame on the FAST beamline. (*c*) Stress curve during creep loading. The black line represents the macroscopic stress measured by the load cell, while the red line shows the average loading-direction stress obtained from ff-HEDM, weighted by grain volume. Only the portion of the data used in this study is shown.

The ff-HEDM experiment was conducted on the Forming and Shaping Technology (FAST) beamline of the Cornell High Energy Synchrotron Source (CHESS). The experimental setup is illustrated in Fig. 2 ▸(*a*). A volume of 1 × 1 × 1 mm was illuminated with an X-ray energy of 61.332 keV with an energy bandwidth of 0.05%. A monochromatic parallel beam was used and the beam size was 1 × 2.5 mm (horizontal × vertical), set by two pairs of tungsten blades acting as slits located about 2 m upstream of the sample. The beam divergence was 0.05 × 0.01 mrad (horizontal × vertical). Diffraction images were collected using the dual-panel Dexela 2923 detector located 1.384 m from the sample. For each ff-HEDM measurement, the sample was rotated 360° about the vertical loading axis, with images integrated over a step size of 0.25°.

**Figure 3:**
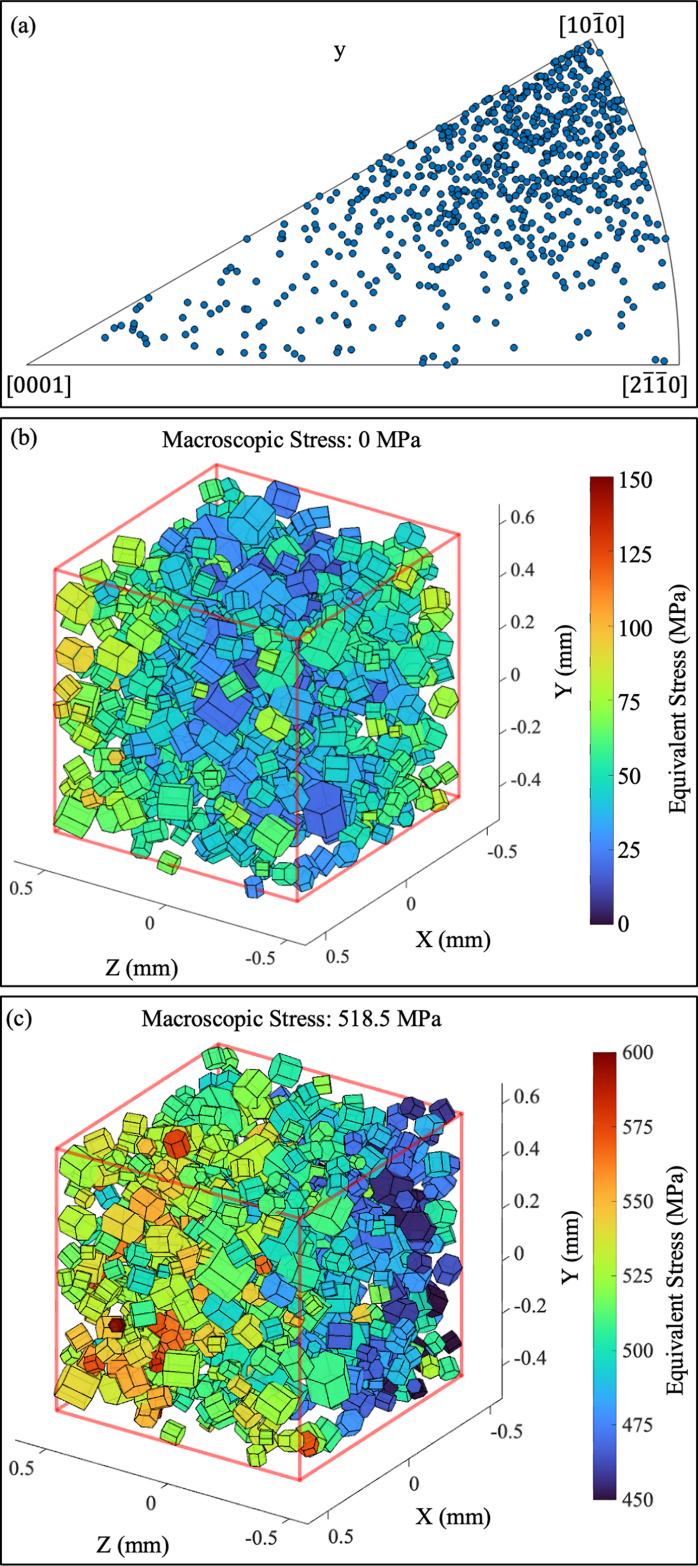
The ff-HEDM reconstruction. (*a*) Inverse pole figure showing orientations of the 752 grains used in analysis, with the IPF axis aligned with the loading direction. (*b*) and (*c*) Three-dimensional visualizations of the indexed grains at unload and at the first measurement during creep, respectively. Each hexagonal prism represents a grain, with prism size indicating relative volume, orientation indicating crystallographic orientation and color indicating equivalent stress. The stress gradient arises from a small bending moment imparted during sample gripping.

The RAMS [rotational and axial motion system, shown in Fig. 2 ▸(*b*)] load frame (Shade *et al.*, 2015 ▸) was used in the experiment, enabling sample rotation during loading so that ff-HEDM data could be collected *in situ*. In the uniaxial tensile creep test, the sample was initially loaded to 85% of its yield strength (513.3 MPa) and held at this load for 22 h. Subsequently, the load was increased to 90% of the yield strength for 7 h. The ff-HEDM measurements were taken every 10 min throughout the experiment. Fig. 2 ▸(*c*) shows the loading curve. The ff-HEDM stress appears slightly higher than the macroscopic stress. This discrepancy, as well as the nonzero stress observed in some grains during the unload step [Fig. 3(*b*)], may be attributed to additional force applied by the acoustic emission sensors attached to the sample. However, the acoustic emission data were too noisy to yield meaningful insights and are therefore not included in this study.

## 3. Methods

The ff-HEDM data were processed and reconstructed using the *HEXRD* software package (Bernier *et al.*, 2024 ▸; Avery *et al.*, 2025 ▸). First, the detector configuration was calibrated as described in Appendix *A*1. Then, from each ff-HEDM measurement, the centroid, grain-averaged crystallographic orientation and grain-averaged elastic strain tensor were indexed and the relative volume was subsequently calculated for each grain, as shown in Fig. 3 ▸. The volume calculation procedure is detailed in Appendix *A*2.

Across the two creep periods analyzed in this study, a total of 1084 grains were indexed. Of these, 752 grains were successfully tracked with a completeness greater than 0.96 and a χ^2^ value less than 0.001. Completeness refers to the ratio of the detected number of diffraction peaks to the expected number of peaks, while χ^2^ serves as an error metric indicating the goodness of fit. These metrics are explained in greater detail by Bernier *et al.* (2011 ▸). As shown by the inverse pole figure (IPF) in Fig. 3 ▸(*a*), the material exhibits weak texture.

In this section, we describe the four methods for detecting grain-scale plastic deformation events from ff-HEDM measurements: (1) equivalent stress relaxation, (2) RSS relaxation, (3) orientation change and (4) diffraction peak shape change. In this work, Methods 3 and 4 are used to cross-validate the results obtained from Methods 1 and 2. The latter exhibit more gradual and noise-sensitive responses in our setup, making it difficult to determine robustly the onset and duration of plastic deformation; however, they provide valuable supporting evidence. To illustrate the application of all four methods, three randomly selected grains, labeled Grains A, B and C, are presented as representative examples of different event types: Case 1, Case 2 and no event, respectively. The definitions of Case 1 and Case 2 events will be introduced in the following section.

### 3.1. Method 1 – equivalent stress relaxation

Analogous to the stress relaxation observed on the macroscale in many metallic materials, known as the yield point phenomenon (Hall, 2012 ▸), grain-scale equivalent stress relaxation can serve as an indicator of plastic events on the grain level. This method was demonstrated by Beaudoin *et al.* (2017 ▸) for Ti–7Al alloys; in that case, decreasing equivalent stresses were required to occur across at least two sequential ff-HEDM measurements.

In this work, the grain-averaged stress tensor is calculated by multiplying the grain-averaged elastic strain tensor with the stiffness tensor provided in Table 1 ▸ (Lim *et al.*, 2021 ▸), as described by 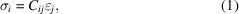
where **σ** and **ɛ** are in Voigt notation and 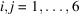. The von Mises (subscript VM) equivalent stress is then calculated using 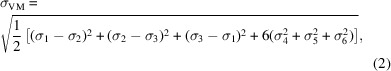
where σ_*i*_ is in Voigt notation.

An equivalent stress relaxation event (corresponding to a plastic event) is defined by the magnitude and duration of the stress decrease or relaxation. In our experiment, two distinct types of stress relaxation events were observed: (i) a sharp relaxation that occurred over a short period of time, referred to as Case 1 events, and (ii) a gradual relaxation that occurred over a longer period of time, referred to as Case 2. Given that the strain resolution of ff-HEDM is 10^−4^ (Bernier *et al.*, 2011 ▸; Park *et al.*, 2017 ▸) and Young’s modulus of the material is 110 GPa (Radecka *et al.*, 2016 ▸), the initial threshold for equivalent stress relaxation was set to 11 MPa to distinguish true relaxation events from measurement noise. Through iterative refinement, this threshold was adjusted slightly as described below.

**Figure 4:**
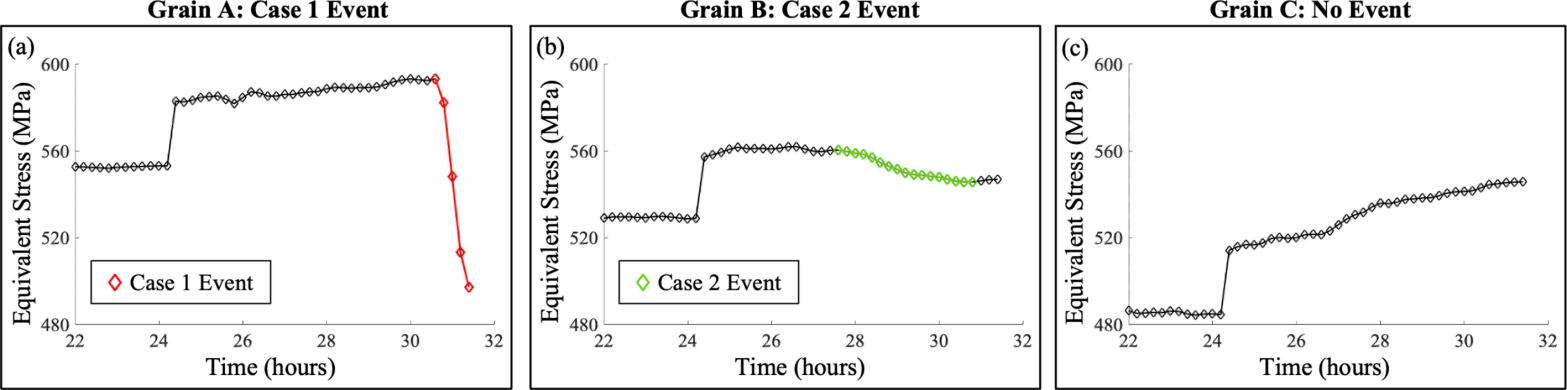
Method 1: equivalent stress relaxation. Grains A, B and C are examples for (*a*) a Case 1 event detected, (*b*) a Case 2 event detected and (*c*) no event detected, respectively. Detected events are highlighted in red for Case 1 and green for Case 2. Each diamond marker corresponds to one time-resolved ff-HEDM measurement.

For Case 1 events, the minimum (threshold) equivalent stress relaxation magnitude required to identify a plastic event σ_VM,th1_ was set to 12.5 MPa for the total relaxation magnitude of the event and the minimum relaxation duration *T*_VM,th1_ was set to two consecutive measurements (which is approximately 20 min in this experiment). For Case 2 events, the total relaxation magnitude was generally smaller than that of Case 1 events, while the relaxation duration was much longer. Thus, the minimum total relaxation magnitude to identify a Case 2 plastic event σ_VM,th2_ was set to 6 MPa and the minimum relaxation duration *T*_VM,th2_ was four consecutive measurements (40 min). In other words, the magnitude threshold was halved and the duration threshold was doubled for Case 2 relative to Case 1. Although the relaxation magnitude threshold for Case 2 is smaller than the strain resolution, a consistent decrease in equivalent stress over four or more measurements signifies plastic events rather than random noise. Examples of grains with detected Case 1 events, Case 2 events and no detected events are shown in Fig. 4 ▸.

### 3.2. Method 2 – RSS relaxation

When a grain undergoes plastic deformation, it is expected to experience a decrease in RSS on the active slip system(s). For example, Worsnop *et al.* (2022 ▸) demonstrated that grain-averaged RSS values can either increase or decrease during creep loading. While an increase in RSS may result from the redistribution of stress across the polycrystalline grain network, a decrease is indicative of plastic deformation occurring within the grain.

Using the elastic strain tensor and the orientation measured with ff-HEDM, the RSS on a specific slip system is calculated as **τ** = *b*_*i*_σ_*ij*_*n*_*j*_, where **b** is the Burgers vector, **n** is the slip-plane normal of the slip system and **σ** is the stress tensor. In this study, we consider the basal, prismatic and pyramidal 〈*a*〉 slip systems. The critical RSS (CRSS) of each slip system at room temperature is shown in Table 2 ▸ (Pagan *et al.*, 2018 ▸).

Similarly to Method 1 in Section 3.1, an RSS relaxation event corresponding to a plastic event is defined by the magnitude of the RSS decrease or relaxation and the duration of the relaxation event. Like with Method 1, two types of relaxation were observed for Method 2: Case 1 (sharp decrease, short duration) and Case 2 (gradual decrease, longer duration) events. In general, the RSS values on slip systems are lower than the equivalent stress values; thus, the relaxation magnitudes on slip systems are correspondingly smaller. The criteria for Method 2 were primarily determined by observations of the RSS versus time graphs and refined through trial and error.

For Case 1 events, the minimum relaxation magnitude to identify a plastic event σ_RSS,th1_ was set to 9 MPa. To distinguish possible sudden drops introduced by noise, the minimum relaxation duration required to identify a plastic event *T*_RSS,th1_ was two consecutive measurements (20 min). For Case 2 events, the magnitude threshold σ_RSS,th2_ was set to 4.5 MPa and the duration threshold *T*_RSS,th2_ was four continuous measurements (40 min).

**Figure 5:**
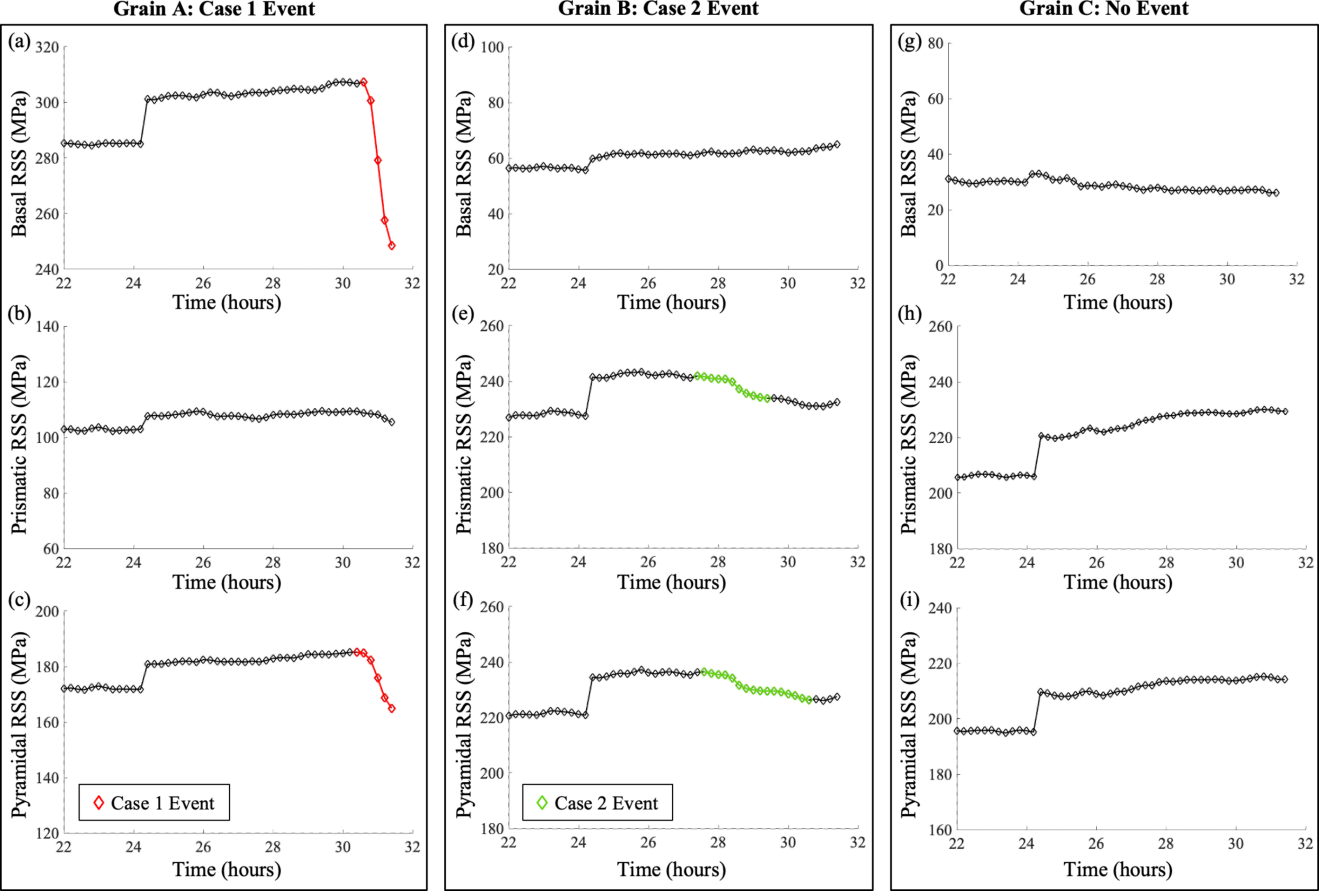
Method 2: RSS relaxation. Grains A, B and C are used as examples for (*a*)–(*c*) a Case 1 event detected, (*d*)–(*f*) a Case 2 event detected and (*g*)–(*i*) no event detected, respectively. For each grain, three slip systems are considered, namely basal, prismatic and pyramidal 〈*a*〉 (from top to bottom, respectively). Detected events are highlighted in red for Case 1 and green for Case 2. Each diamond marker corresponds to one time-resolved ff-HEDM measurement.

Additionally, to identify a plastic event associated with a specific slip system, the local RSS must exceed the CRSS for that slip system. Here, ‘local’ refers to the region within the grain where the plastic event occurs. However, since ff-HEDM provides grain-averaged information, the calculated RSS represents an average RSS of the slip system over the entire grain. To account for this, a scale factor of 0.65 is applied to the CRSS for each slip system. The observed RSS value must exceed this adjusted CRSS value for a relaxation to be classified as a plastic event. Fig. 5 ▸ shows examples of plastic events identified by RSS relaxation. Notably, grains A and B in Fig. 5 ▸ exhibit relaxation events on two slip systems, suggesting the potential activation of multiple slip systems during deformation.

### 3.3. Method 3 – orientation change

When plastic deformation events occur, the local (*i.e.* intragranular) orientation changes as a result, leading to a cumulative change in the grain-averaged orientation. This phenomenon has been used to detect and analyze grain-scale plasticity from ff-HEDM measurements, as demonstrated by Lim *et al.* (2022 ▸) for Ti–7Al alloys during cyclic loading. The orientation resolution of the experiment was 0.01° (Bernier *et al.*, 2011 ▸; Park *et al.*, 2017 ▸). In our measurements, we found that the incremental orientation change was smaller than, or of the order of, the orientation resolution. As a result, it was difficult to determine specific change points corresponding to when a plastic event started and ended. For this reason, grain-scale orientation change was used for cross-validation of Methods 1 and 2.

**Figure 6:**
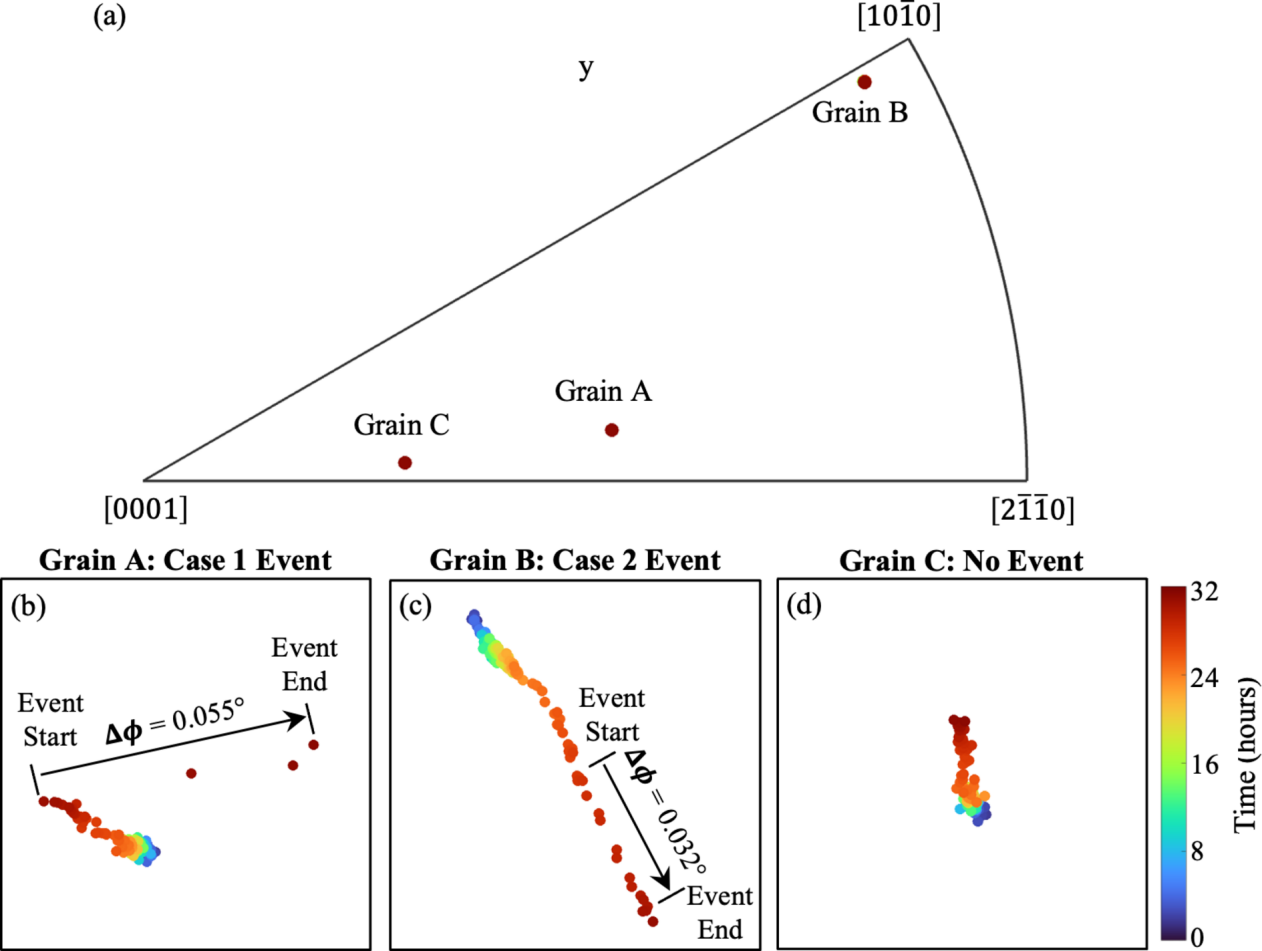
Method 3: orientation change. Validation for grain-scale plastic events detected using Methods 1 and 2 in Figs. 4 and 5, where Grains A, B and C show examples of Case 1, Case 2 and no event, respectively. (*a*) Orientations of Grains A, B and C on an IPF aligned with the loading direction. (*b*)–(*d*) The orientation of each grain during creep loading in enlarged subsets of the IPF. Each marker represents an ff-HEDM measurement and is colored by the loading time. Δϕ is the total orientation change from before to after an event.

For each grain-scale plastic event identified via Methods 1 or 2, we assessed the orientation change between before and after the event. To minimize biases caused by individual measurement errors, the ‘before’ orientation was defined as the mean orientation from the three ff-HEDM measurements preceding the event start, and the ‘after’ orientation was defined as the mean orientation from the three ff-HEDM measurements following the event end. If the difference between these two mean orientations, Δϕ, exceeded 0.02° (twice the orientation resolution), then we would consider this as a validation of the detection of a grain-scale plastic deformation event. This is demonstrated in Fig. 6 ▸.

### 3.4. Method 4 – diffraction peak shape change

When a grain undergoes plastic deformation, plastic strain gradients can lead to the accumulation of GNDs, which manifest as orientation gradients within the grain, as discussed in Section 3.3. These orientation gradients result in diffraction peak broadening, particularly in the η and ω directions [see Fig. 1 ▸(*d*)]. This phenomenon has long been recognized, and prior studies (Ungár *et al.*, 2010 ▸; Borbély & Ungár, 2012 ▸) have provided theoretical insight into the relationship between diffraction broadening and dislocation structures. With the advancement of ff-HEDM techniques, changes in diffraction peak shape have been used to detect grain-scale plastic deformation, as demonstrated in prior studies (Chatterjee *et al.*, 2019 ▸; Wang *et al.*, 2021 ▸; Pagan & Miller, 2014 ▸; Pagan & Miller, 2016 ▸).

**Figure 7:**
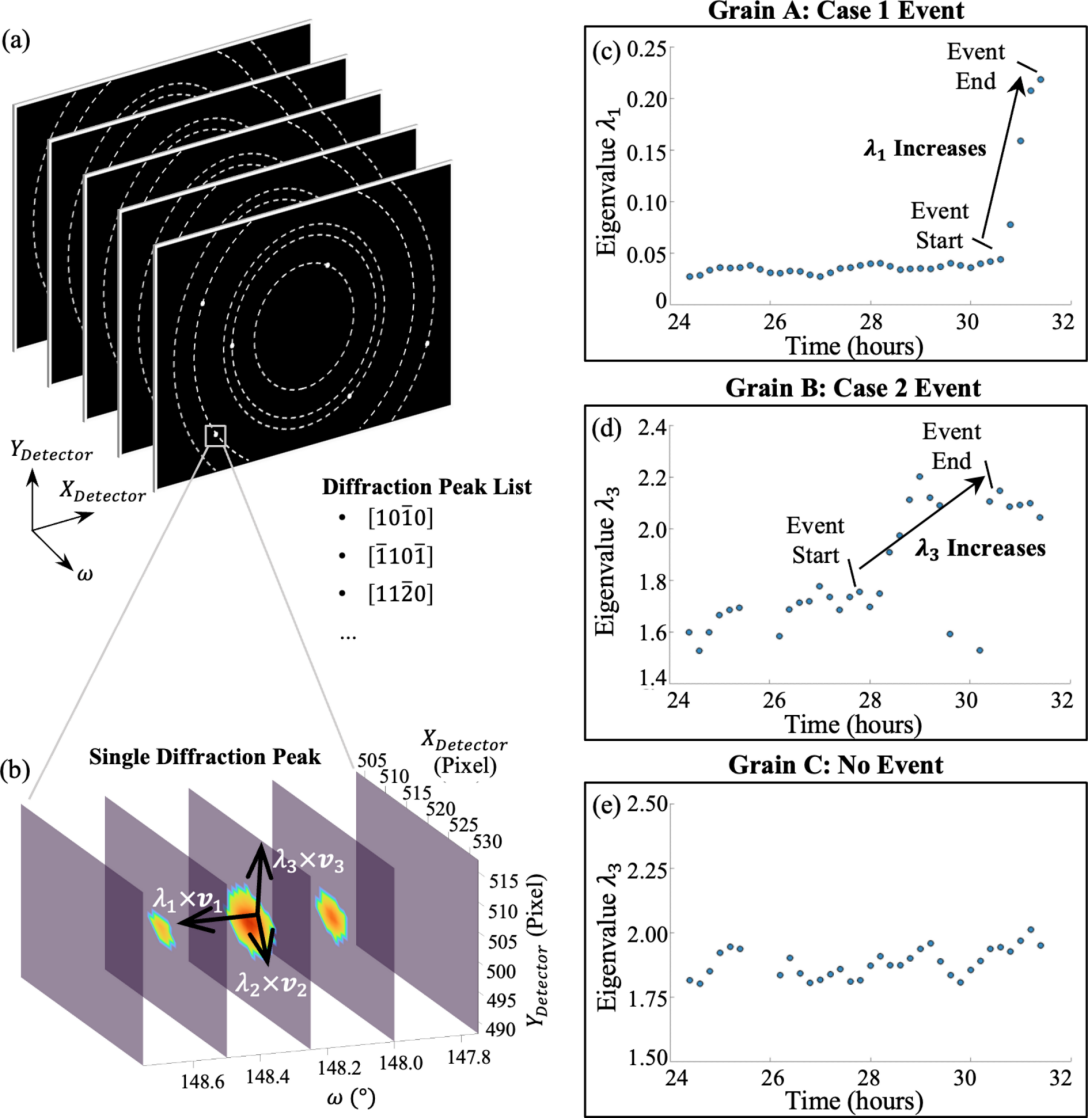
Method 4: diffraction peak shape change. Validation for grain-scale plastic events detected using Methods 1 and 2 in Figs. 4 and 5, where Grains A, B and C show examples of Case 1, Case 2 and no event, respectively. (*a*) Each grain analyzed will form different diffraction peaks at different angles for different crystal planes. (*b*) Each diffraction peak can be described as all the illuminated pixels in the 3D space defined by the detector *x* and *y* directions and ω, the rotation direction. The shape of the peak can be characterized by three eigenvalues (λ_*i*_) and eigenvectors (*v*_*i*_) obtained from PCA. (*c*)–(*e*) During plastic events, eigenvalues increase, indicating peak broadening. Shown here is the eigenvalue change for the 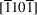 diffraction spot for Grains A, B and C.

For Method 4, we use the following procedure for every grain identified with plastic event(s) by Method 1 or Method 2. In ff-HEDM data analysis, every indexed grain is associated with hundreds of diffraction peaks, each corresponding to a distinct *hkl* reflection. These diffraction peaks exist in a three-dimensional space defined by the detector plane (*X*_Detector_, *Y*_Detector_) and the sample rotation angle (ω). For each diffraction peak, a 3D subvolume centered on the peak was first extracted in the (*X*_Detector_, *Y*_Detector_, ω) space from the raw ff-HEDM data set. Background noise within this cropped region was removed (detailed procedures are in shown in Appendix *A*3) and the peak was centered using its intensity-weighted centroid. To quantify the shape of each diffraction peak, a weighted principal component analysis (PCA) was applied, where the intensity served as the weighting factor. The resulting eigenvalues (λ_1_, λ_2_, λ_3_) and eigenvectors (*v*_1_, *v*_2_, *v*_3_) describe the shape of the peak in 3D space, as illustrated in Fig. 7 ▸(*b*). The eigenvector associated with the smallest eigenvalue indicates the direction of least intensity spread, while the eigenvector corresponding to the largest eigenvalue denotes the direction of greatest spread.

The detailed PCA calculation is as follows. For each diffraction peak, an *n* × 3 matrix **X** was constructed, where each row contains the coordinates (*x*_*i*_, *y*_*i*_, ω_*i*_) of an illuminated pixel. A diagonal *n* × *n* matrix **W** was also defined, where each *w*_*i*_ represents the intensity of pixel (*x*_*i*_, *y*_*i*_, ω_*i*_): 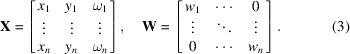
In both cases, *n* is the total number of pixels. The covariance matrix **C** was then computed as
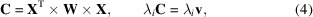
with subsequent eigendecomposition to obtain the eigen­values and eigenvectors, which were then used to track diffraction peak shape changes.

Given the detector resolution and rotation step size in this experiment (0.25°), the detection of diffraction peak shape changes was limited and significantly influenced by noise, making it challenging to determine the exact onset of eigenvalue change points (*i.e.* when plasticity events started and ended). However, it was still possible to observe that, when a plastic event occurred in the grain, the eigenvalue along certain directions increased, indicating peak spreading. Therefore, for each event identified by Methods 1 or 2, Method 4 was used for cross-validation. Specifically, the mean eigenvalues before and after the event were calculated and compared. If the difference between the two average eigenvalues exceeded twice the standard deviation, the diffraction peak shape was considered to have changed. For example, in Grain A shown in Fig. 7 ▸(*c*), λ_1_ increased sharply during the detected event, indicating that the diffraction peak has spread along the rotation direction. In Grain B shown in Fig. 7 ▸(*d*), λ_3_ increased, reflecting spreading on the detector plane.

As mentioned above, every grain has multiple diffraction peaks. After analyzing the shape evolution of all diffraction peaks for a given grain, if more than 20% of the diffraction peaks exhibited shape changes during the plastic event, we considered the event validated.

Diffraction peaks are generally better described in η–2θ–ω space, as this coordinate system is directly related to the reciprocal-lattice vector, whereas the *X*–*Y* coordinates represent a projection onto the detector plane. In this study, we chose to work in *X*–*Y*–ω space, because the diffraction peak shape point clouds were typically small and symmetric (due to the equiaxial well annealed grain structure), resulting in similar PCA trends between the two coordinate system choices. In this case, the *X*–*Y* representation allows for a more straightforward analysis that can be performed directly from the detector images. However, if larger, more spread, diffraction peaks or higher-resolution measurements are available, then peak shape analysis in η–2θ–ω space may be preferable.

## 4. Results

In this study, we explored four methods (see Section 3) to identify grain-scale plastic deformation events using time-resolved ff-HEDM measurements. A significant challenge in this analysis is the lack of ground truth for 3D grain-scale plasticity events during mechanical loading, as no simultaneous independent experimental confirmation is available. To address this limitation, we employ cross-validation using the four different detection methods.

### 4.1. Identification of grain-scale plastic deformation events

During the two creep periods analyzed in this study (22 h at 85% of the yield strength and 7 h at 90% of the yield strength), a total of 208 grain-scale plasticity events were detected using Method 1, 288 events were detected using Method 2, and 137 events were detected using both Methods 1 and 2 (Table 3 ▸). Specifically, during the first creep loading period, 86 grain-scale plasticity events were detected with Method 1, 122 with Method 2, and 53 with both Methods 1 and 2. During the second creep loading period, 122 grain-scale plasticity events were detected with Method 1, 166 with Method 2, and 84 with both Methods 1 and 2.

As described in Sections 3.1 and 3.2, the detected events were categorized into two types. Of the 208 total events detected with Method 1, 128 were classified as Case 1 and 80 were classified as Case 2. Of the 288 events detected with Method 2, 98 were classified as Case 1 and 190 were classified as Case 2 (Table 4 ▸).

### 4.2. Cross-validation of detected plastic events

**Figure 8:**
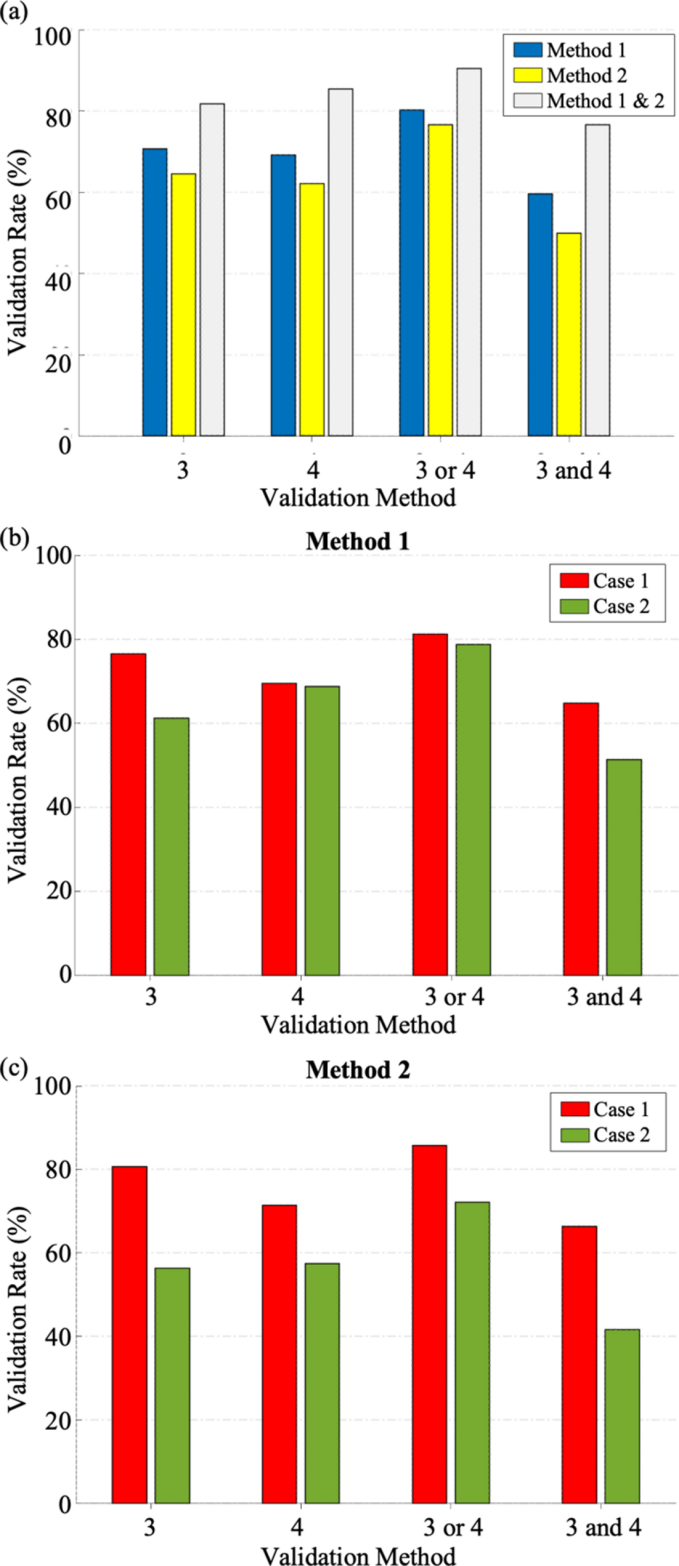
Validation rates for (*a*) grain-scale plastic events identified by Method 1, Method 2, and both Methods 1 and 2, (*b*) Case 1 and Case 2 events identified by Method 1, and (*c*) Case 1 and Case 2 events identified by Method 2. Validation was performed using Method 3 and Method 4.

After identifying grain-scale plastic deformation events with Method 1 and Method 2, we cross-validated the detected events using Method 3 and/or Method 4. The cross-validation results are summarized in Table 3 ▸ and Fig. 8 ▸(*a*). Comparing Methods 1 and 2, Method 2 detects more events (288 for Method 2 versus 208 for Method 1), while Method 1 exhibits higher validation rates. Specifically, 71% of the events detected using Method 1 were validated using Method 3 (versus 65% for Method 2) and 69% of the events detected using Method 1 were validated using Method 4 (versus 62% for Method 2). This result suggests that Method 1 may be less prone to false positives than Method 2. However, when cross-validated by either Method 3 *or* Method 4, the difference between Methods 1 and 2 slightly decreased, with 80% of events validated for Method 1 and 77% for Method 2. Regarding cross-validation methodologies, both Method 3 and Method 4 achieved comparable validation rates, with Method 3 showing a slightly higher validation rate for both Method 1 and Method 2. Notably, 91% of the 137 events detected by both Methods 1 *and* 2 were confirmed by cross-validation using Methods 3 *or* 4, suggesting that the plastic deformation events that could be detected by both Method 1 *and* Method 2 were the most reliable.

Table 4 ▸ and Figs. 8 ▸(*b*) and 8 ▸(*c*) show the cross-validation rates for Case 1 and Case 2 events detected using Methods 1 and 2. Both Methods 1 and 2 exhibited high validation rates for Case 1 events (81% when validated using either Method 3 or Method 4), suggesting that Case 1 events are easier to detect and/or validate. In contrast, Case 2 events generally showed slightly lower validation rates (79% when validated using either Method 3 or Method 4). The Case 2 events detected using Method 2 exhibited the lowest cross-validation rate (72% when validated using either Method 3 or Method 4).

Regarding cross-validation methodologies, Method 3 consistently showed higher validation rates for Case 1 events than Method 4, regardless of the identification method used. Additionally, Method 3 exhibited a notably large difference in validation rates between Case 1 and Case 2 events for both identification methods.

### 4.3. Spatial and temporal distribution of plastic events

**Figure 9:**
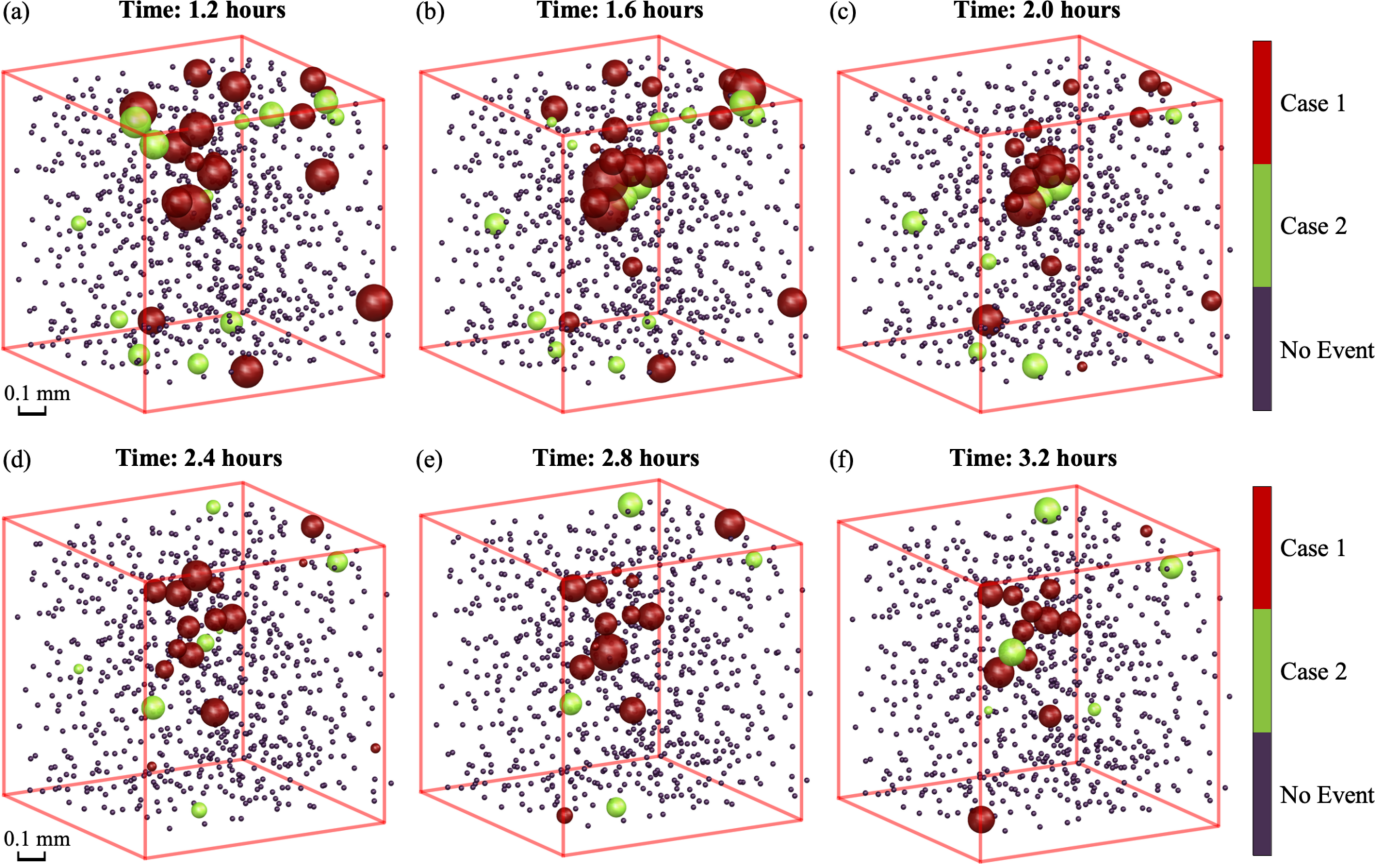
Visualization of the grain-scale plastic events characterized by Method 1 and validated by Method 3 or 4 at (*a*) 1.2 h, (*b*) 1.6 h, (*c*) 2 h, (*d*) 2.4 h, (*e*) 2.8 h and (*f*) 3.2 h after the first creep period (85% of yield strength) started. Each marker here represents a grain and it is sized by the magnitude of the decrease in equivalent stress. The markers are colored red for Case 1 events, green for Case 2 events and blue for no event.

The grain-scale plastic deformation events detected by Method 1 and validated by Method 3 or 4 are visualized as a function of time in the video provided in the supporting information and in Fig. 9 ▸, as Method 1 exhibits a slightly higher validation rate. Fig. 9 ▸ shows a series of snapshots during the first creep period (at 85% yield strength), while the video encompasses both creep periods (at 85% and 90% yield strength). In both the video and Fig. 9 ▸, each marker represents a grain. The markers are positioned at the measured grain centroid, the marker color indicates whether a Case 1 or Case 2 event is being detected (or no event), and the marker size corresponds to the magnitude of the equivalent stress relaxation.

A ‘burst’ of grain-scale plastic deformation events can be observed at the onset of each creep period (at 1.8 h for 85% stress and 24.4 h for 90% stress). Fewer events occur in the latter half of the first creep period, indicating a relatively ‘quiet’ state. Other observations include the fact that many events, especially during the first creep period, occur mainly near the sample surfaces. In contrast, in the second creep period, more plastic events are observed and more events are found with grains closer to the sample center.

What is also apparent, especially from the video, is that plastic events in one grain appear to ‘trigger’ plastic events in neighboring grains, causing chain reactions of grain-scale plasticity that propagate through the 3D grain network. As a result, grain-scale plastic deformation events often appear in clusters that dissipate outwards in space and time. One such cluster is shown in Fig. 9 ▸. Furthermore, Case 1 events are frequently observed at the center of these clusters, while Case 2 events are often observed near the edges (*i.e.* around the Case 1 events). Similar behavior was observed by previous researchers using the equivalent stress relaxation detection method (Method 1) (Beaudoin *et al.*, 2017 ▸). The characterized grain-scale plastic events shed light on many underlying mechanisms that require further study, including (i) when and where these events start, (ii) how these events influence deformation in the neighboring grains, and (iii) what causes the two different types (Case 1 and Case 2) of events.

## 5. Discussion

### 5.1. Pros and cons of Method 1 versus Method 2 for detection

Comparing the two detection approaches, Method 1 and Method 2, each method offers different advantages and limitations. Both methods are straightforward in terms of calculation and implementation. Both equivalent stress and RSS can be calculated in an uncomplicated manner using the grain-averaged elastic strain tensor and, in the case of RSS, the crystallographic orientation. Method 1 demonstrates a slightly higher validation rate than Method 2, suggesting that it is better at distinguishing grain-scale plasticity events from noise. However, this increased validation rate comes at the cost of a lower detection rate, since Method 1 detects fewer total events than Method 2. In particular, Method 1 detects significantly fewer Case 2 events (classified by smaller, more gradual, stress relaxations over longer periods of time) than Method 2.

Method 2’s enhanced detection rate, especially for Case 2 events, can probably be attributed to the use of RSS instead of equivalent stress. In RSS calculations, the grain-averaged stress tensor obtained via ff-HEDM is resolved onto specific slip planes and directions, reducing the stress tensor to a scalar value that encapsulates the driving force needed for plastic deformation. In other words, RSS is more directly related to plastic deformation on a specific slip-system family than equivalent stress. As a consequence, RSS is potentially more sensitive to the changes (*i.e.* relaxations) involved in plastic deformation events. Additionally, the smaller magnitude of RSS values compared with equivalent stress necessitates a lower threshold for identifying relaxation events. Although this lower threshold enables Method 2 to detect finer-scale relaxations, it simultaneously increases its possibility of mistaking noise for detected events, *i.e.* introducing false positives. In contrast, Method 1’s higher threshold criterion results in reduced sensitivity to minor stress relaxations but provides greater robustness against noise, explaining its higher validation rates.

Another advantage of Method 2 is the potential to use it to identify the specific slip-system family or families that are activated during the grain-scale plastic deformation events – information that Method 1 does not directly provide. This capability is especially useful when slip occurs on multiple slip-system families simultaneously. For example, for Grain A in Fig. 5 ▸, Case 1 plastic deformation events are detected from RSS relaxation for basal slip [Fig. 5 ▸(*a*)] and pyramidal slip [Fig. 5 ▸(*c*)] but not for prismatic slip [Fig. 5 ▸(*b*)]. These results may indicate that basal slip and pyramidal slip are both occurring inside the grain. Similarly, for Grain B in Fig. 5 ▸, Case 2 plastic deformation events are detected from RSS relaxation for prismatic slip [Fig. 5 ▸(*e*)] and pyramidal slip [Fig. 5 ▸(*f*)] but not basal slip [Fig. 5 ▸(*d*)], suggesting that prismatic slip and pyramidal slip are both occurring inside the grain. Thus, Method 2 may offer more detailed insights into grain-scale plastic deformation.

However, Method 2 inherently assumes that plastic deformation occurs through slip on specific slip systems (*i.e.* basal, prismatic, pyramidal 〈*a*〉 *etc.*). In this study we use a Ti–7Al sample, a material known to deform primarily through crystallographic slip, with no evidence of twinning or other competing mechanisms under our experimental conditions. Therefore, we consider the assumption of slip-dominated deformation to be valid in this case. That said, Method 2 would not be appropriate for materials where deformation modes cannot be ruled out that do not obey an RSS criteria and/or do not have known CRSS values. In such cases, Method 1 – which does not rely on slip system assumptions – would remain applicable.

### 5.2. Categorization of plastic events (Case 1 versus Case 2)

As introduced in Sections 3.1 and 3.2, the grain-scale plasticity events detected in this study can be categorized into two types. As shown in Table 4 ▸, more Case 1 events are detected using Method 1, whereas more Case 2 events are detected using Method 2. This trend holds true even when considering only validated events (using either validation method). As discussed in the previous section, due to the higher sensitivity of Method 2 and the subtle nature of Case 2 events, it is likely that many Case 2 events go undetected by Method 1 because of their smaller magnitude.

**Figure 10:**
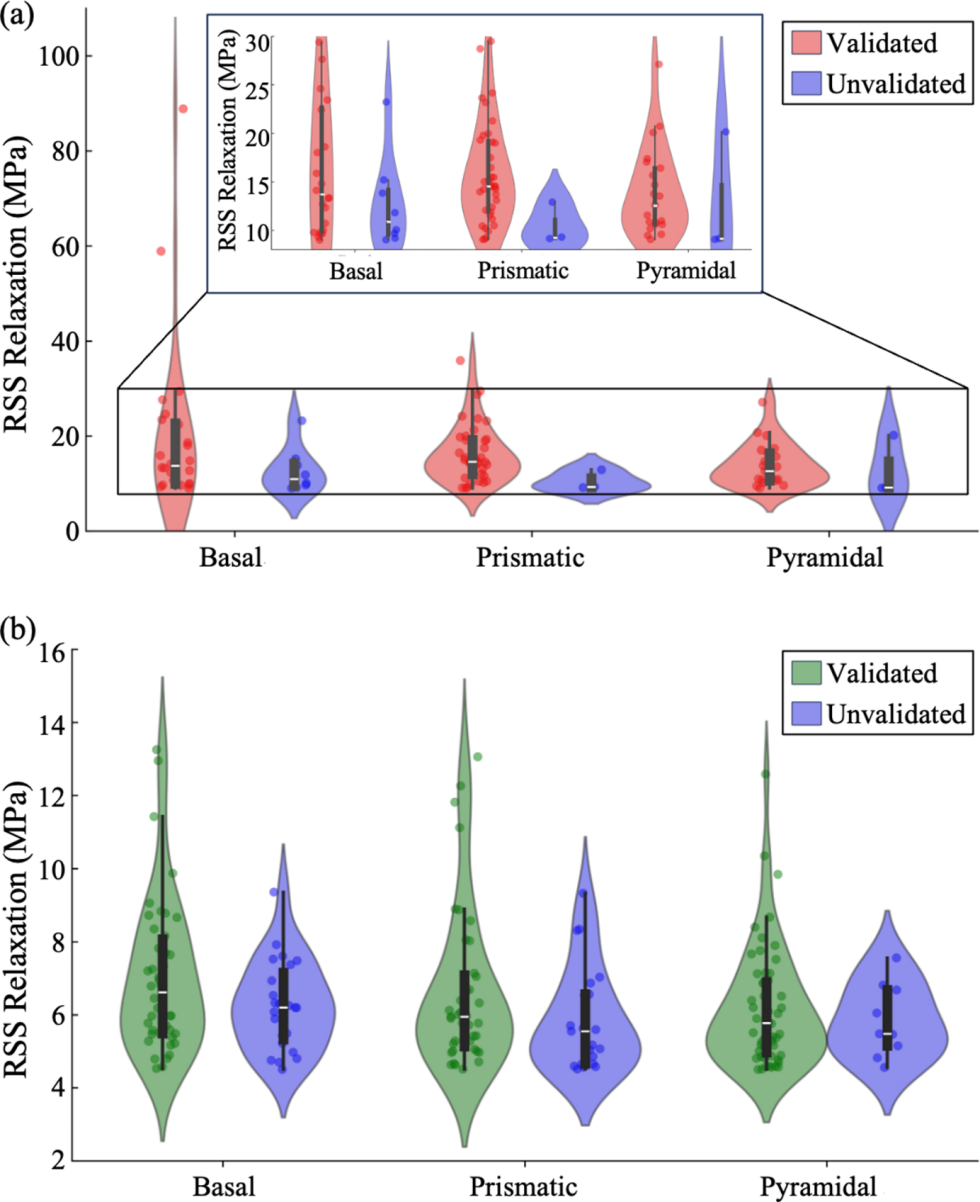
RSS relaxation magnitude distribution of (*a*) Case 1 and (*b*) Case 2 grain-scale plastic events identified by Method 2, categorized by primary slip system, defined as the slip system with the maximum RSS versus CRSS ratio. Red and green markers denote validated Case 1 and Case 2 events, respectively, whereas blue markers indicate unvalidated events.

Since Method 2 offers higher sensitivity and can directly identify the activated slip system(s), we categorized the events detected by Method 2 by their primary slip system – defined as the slip system with the highest RSS versus CRSS ratio among the activated slip families – to understand better the differences between Case 1 and Case 2 events, as shown in Fig. 10 ▸. Figs. 10 ▸(*a*) and 10 ▸(*b*) show the RSS relaxation magnitude distributions for Case 1 and Case 2 events identified by Method 2, respectively. The distributions are presented as violin plots, with events grouped by slip-system families and further separated into validated (by Method 3 or Method 4) and unvalidated events. The width of each violin indicates the distribution density, while the height indicates the magnitude of the RSS relaxation used to detect the event using Method 2. The black box inside each violin denotes the interquartile range, with a white line indicating the median. The largest relaxation magnitudes are associated with basal slip, while events dominated by pyramidal slip exhibit smaller relaxation magnitudes. The majority of unvalidated events correspond to lower relaxation magnitudes.

**Figure 11:**
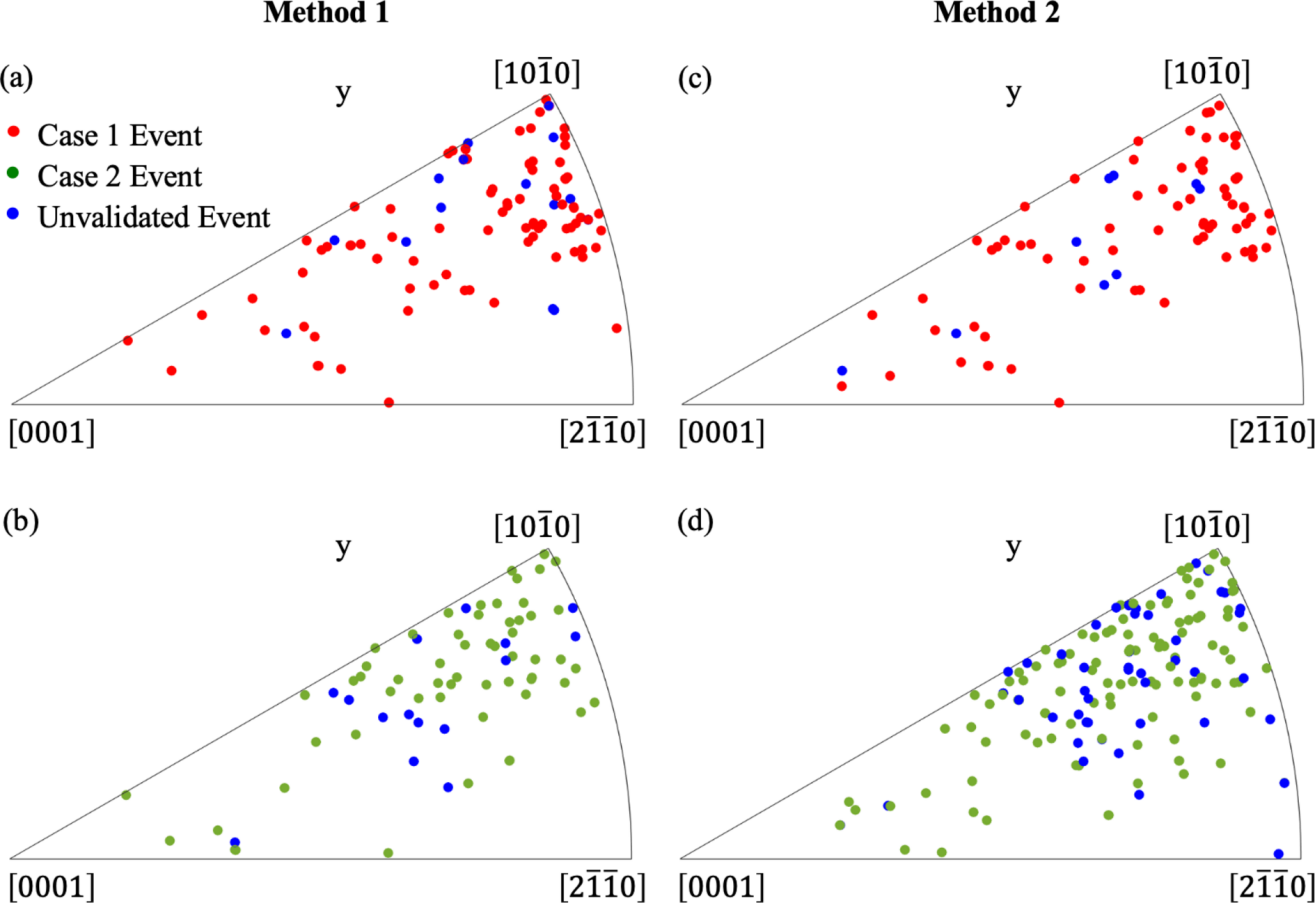
IPF showing the orientations of grains undergoing (*a*) Case 1 and (*b*) Case 2 events identified by Method 1, and (*c*) Case 1 and (*d*) Case 2 events identified by Method 2. Red markers indicate grains associated with Case 1 events, green markers indicate Case 2 events and blue markers represent unvalidated events.

Table 5 ▸ also summarizes the number of validated and unvalidated Case 1 and Case 2 events identified by Methods 1 and 2 categorized by the primary slip-system families. One notable observation from Table 5 ▸ and Fig. 10 ▸ is that Case 1 events predominantly involve prismatic slip, whereas Case 2 events are more frequently associated with pyramidal 〈*a*〉 slip. In α-phase Ti–Al alloys, prismatic and basal slip are generally recognized as the primary slip modes in room-temperature deformation (Bridier *et al.*, 2008 ▸), *i.e.* they are more easily activated under loading. In our sample, more grains are oriented favorably for prismatic slip [as shown in Fig. 3 ▸(*a*)], resulting in a higher number of events where the prismatic system is the primary slip mode, especially for Case 1 events. Interestingly, there are more Case 2 pyramidal events detected than Case 1. The orientation mapping of grains exhibiting Case 1 and Case 2 events, identified by Methods 1 and 2, is shown in Fig. 11 ▸ and further supports this trend: grains involved in Case 1 events are more frequently found in orientations with higher Schmid factors for prismatic slip, while those associated with Case 2 events tend to align with orientations favoring pyramidal 〈*a*〉 slip, as can be inferred from the Schmid factor contours presented by Li *et al.* (2015 ▸). Due to the limited number of grains in our sample that are favorably oriented for basal slip, fewer events are observed with basal activation, as reflected in the density distributions in Fig. 10 ▸. Although fewer events were observed with basal slip as the primary system, these events tend to exhibit larger relaxation magnitudes. The largest grain-scale plastic events are typically associated with basal slip activity.

Events identified with pyramidal 〈*a*〉 slip generally exhibit smaller relaxation magnitudes and account for a significant portion of the validated Case 2 events. This may be attributed to cross-slip between the prismatic and pyramidal 〈*a*〉 systems, as previously observed by Joseph *et al.* (2018 ▸) and Clouet *et al.* (2015 ▸) through TEM studies. Supporting this, Method 2 identified 113 events with more than one activated slip system, of which 91 involved concurrent activation of both prismatic and pyramidal 〈*a*〉 slip. This suggests that the interaction or transition between these two systems may play a key role in facilitating the more gradual lower-magnitude plastic events categorized as Case 2.

### 5.3. Cross-validation using Methods 3 and 4

Cross-validation using Method 3 and Method 4 is essential in this study due to the absence of a ground truth. As shown in Table 3 ▸, Methods 3 and 4 generally produce similar validation rates for events detected by either Method 1 or Method 2, with Method 3 having slightly higher validation rates. However, when validation rates are separated by event type – Case 1 versus Case 2, as shown in Table 4 ▸ – the performance of the two validation methods diverges. Method 3 consistently shows a higher validation rate for Case 1 events than Method 4, whereas Method 4 has a higher validation rate for Case 2 events, particularly those detected by Method 1. Method 3 exhibits a notable decrease in validation rate from Case 1 to Case 2 events for both detection methods, while Method 4 maintains similar validation rates across both cases.

**Figure 12:**
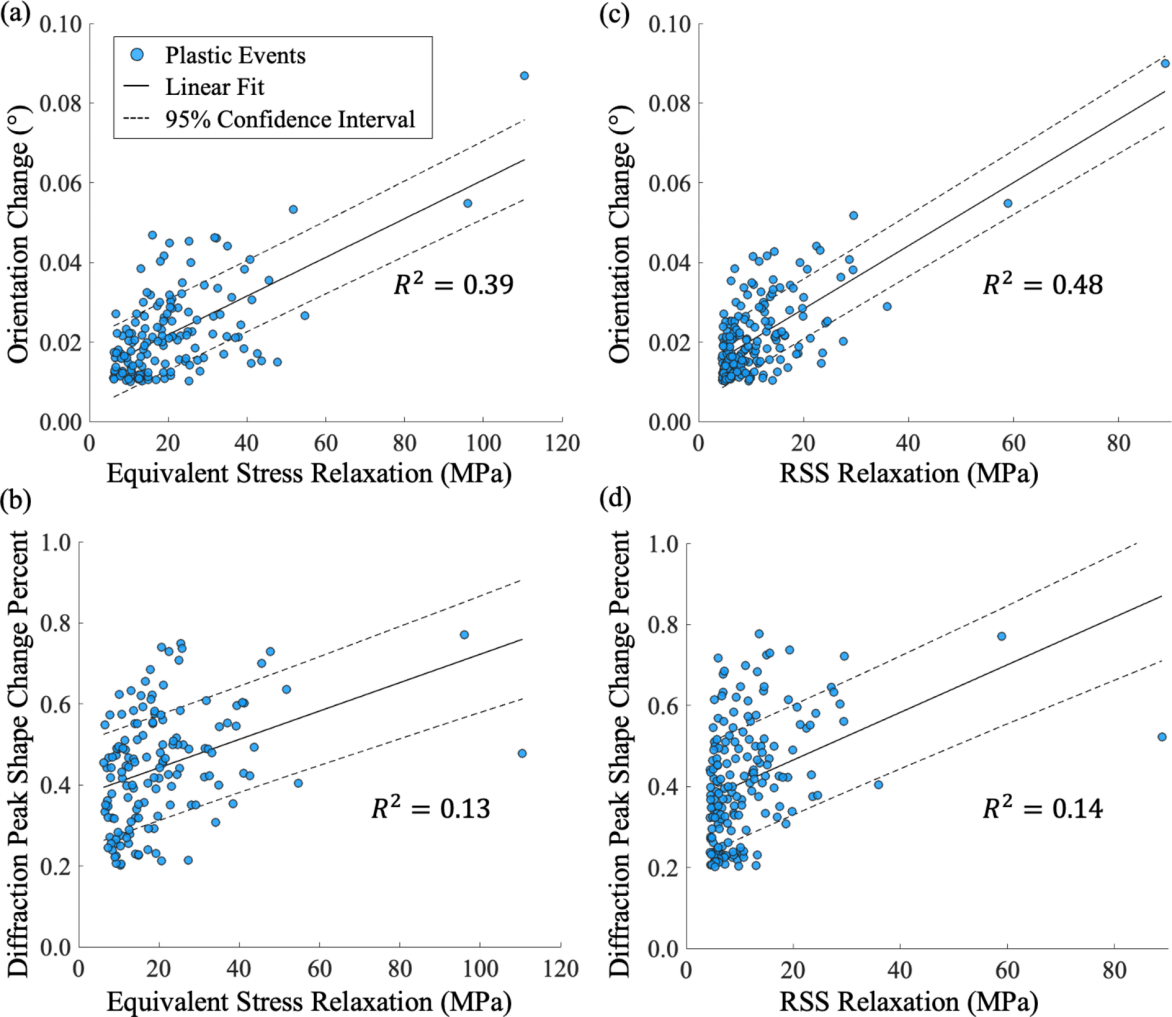
Correlation between event relaxation magnitude and validation metrics for events identified by Method 1 and Method 2. The magnitude of the grain orientation change between before and after the events identified by (*a*) Method 1 and (*c*) Method 2 increases with greater equivalent stress relaxation, indicating a strong correlation between the plastic event magnitude and the lattice reorientation. In contrast, the difference in diffraction peak shape before and after the events identified by (*b*) Method 1 and (*d*) Method 2 exhibits lower sensitivity to the magnitude of stress relaxation than to orientation change.

This trend likely arises because the lattice orientation changes captured by Method 3 are more strongly correlated with the magnitude of stress relaxation than the diffraction peak shape changes measured by Method 4, as suggested in Fig. 12 ▸. Larger stress relaxations are associated with more pronounced plastic deformation and, consequently, more significant lattice reorientations. In contrast, smaller Case 2 events often produce orientation changes that approach or fall below the experimental resolution limit, making them more difficult to validate with Method 3. For Method 4, diffraction peak broadening is expected to increase with larger relaxation magnitudes and greater lattice reorientation, particularly in the azimuthal (η) and rotation (ω) directions. However, due to our experimental setup, with a rotation step size of 0.25°, we were unable to resolve peak broadening adequately in the rotation direction, as most diffraction peaks appeared in only a single frame over the loading period. Fortunately, mis­orientations also lead to broadening in the η direction, to which the current setup is more sensitive. Since each grain produces many Bragg reflections with different orientations, our approach, which analyzes multiple peaks per grain, is still likely to capture broadening effects in reflections where the misorientation projects strongly into the η direction.

In Fig. 10 ▸, the distribution of RSS relaxation magnitudes is shown for events detected by Method 2, separated into valid­ated and unvalidated cases. It is evident that most un­validated events exhibit lower relaxation magnitudes and are therefore more susceptible to being confused with experimental noise. These unvalidated events may represent true but subtle plastic deformation that falls below the detection thresholds of Methods 3 and 4, or they may be false positives resulting from noise in the data. Due to the angular resolution limits of our experimental setup, subtle diffraction peak spreading in the ω direction or small orientation changes may not be fully captured. The use of cross-validation with Methods 3 and 4 improves the robustness of plastic event identification, though it may also filter out some true events along with the noise. Future work with finer ω step sizes and improved detector resolution could enhance the sensitivity of validation methods and enable detection of smaller-scale plastic events with greater confidence.

### 5.4. Implications for future studies

In this work, we have employed a detection–validation process for identifying grain-scale plastic deformation events. Methods 1 and 2 were used for event detection, followed by cross-validation with Methods 3 and 4 due to the absence of a ground truth. This same approach can be applied to future studies of grain-scale plasticity during stress hold periods, such as in creep or dwell fatigue loading, where ff-HEDM captures time-resolved deformation events. In this work, we have demonstrated it with an h.c.p. α-phase Ti–7Al alloy. The same process should be applicable to other alloy systems where slip-related plasticity is of interest.

The effectiveness of this process depends heavily on the quality of ff-HEDM reconstructions, particularly for Methods 1 and 2. Robust and sensitive detection requires data sets with high completeness and low χ^2^ values, as poor indexing can mask true relaxation signals or introduce false positives. For noisy data sets, preprocessing steps (*e.g.* data smoothing) may improve robustness, but these must be applied carefully to avoid smoothing out genuine relaxation events. Additionally, selecting appropriate thresholds for stress relaxation magnitude and duration requires careful calibration. While initial estimates can be based on the resolution limits of the technique, data-set-specific tuning is essential to optimize sensitivity without overcounting transient artifacts.

The sensitivity and robustness of all methods can be further enhanced by improving experimental parameters. A finer rotation step size (ω) increases angular resolution but reduces temporal resolution due to longer scan times. Similarly, a higher detector resolution improves both spatial and angular precision but significantly increases the computational load. With improved measurement resolution, Methods 3 and 4 – currently used for validation – could potentially serve as detection tools themselves in future investigations.

Another important consideration is the time resolution of our experimental setup, which is approximately 10 min between successive ff-HEDM measurements. While the onset of plastic deformation probably occurs on a much shorter timescale, the resulting stress relaxation, orientation changes and diffraction peak shape evolution persist beyond the initial event and remain detectable within this temporal resolution. Although this limits our ability to capture the precise moment of deformation initiation, the lasting signatures enable us to resolve and analyze the consequences of the deformation. With recent advances in ff-HEDM acquisition techniques, significantly faster measurements are now feasible, offering the potential for improved time resolution in future studies. When combined with other microscopy techniques such as near-field HEDM or EBSD measurements, ff-HEDM can provide multi-scale and multi-modal insights that further enhance our understanding of deformation mechanisms.

## 6. Conclusion

In this work, we have evaluated four methods for detecting grain-scale plasticity using time-resolved ff-HEDM measurements: (1) equivalent stress relaxation, (2) RSS relaxation, (3) orientation change and (4) diffraction peak shape change. These methods were applied to an h.c.p. α-phase Ti–7Al alloy under creep loading. As a ground truth was not available, we instead used Methods 1 and 2 for detecting grain-scale plastic events from ff-HEDM data, with Methods 3 and 4 serving as validation. Two types of events were observed: Case 1 events, characterized by large stress relaxations over a short duration, and Case 2 events, which were smaller and more gradual relaxations over a longer duration. Using the validated events, we managed to track the spatial and temporal evolution of grain-scale plasticity during creep loading.

Method 1 exhibited higher validation rates and robustness to noise but was less sensitive to Case 2 events. In contrast, Method 2 captured more subtle events, but this increased sensitivity came at the cost of a lower validation rate. Method 2 also provided additional insights into activated slip systems. For validation, Method 3 showed high agreement with Case 1 events, consistent with the stronger orientation changes induced by larger relaxations. Method 4 validated both Case 1 and Case 2 events at comparable rates. Generally, finer rotation steps and higher detector resolution can improve both sensitivity and robustness, though at the cost of reduced time resolution and increased computational demands. The same detection and validation process can be applied to future studies of grain-scale plastic deformation. In particular, generating synthetic data sets using the crystal plasticity finite element method would be a valuable way to benchmark and validate the performance of the four detection methods.

The distinct characteristics of Case 1 and Case 2 events suggest different underlying mechanisms. Case 1 events were more commonly associated with basal and prismatic slip, particularly the latter, whereas Case 2 events were more frequently linked to pyramidal 〈*a*〉 slip. Given the frequent simultaneous activation of prismatic and pyramidal slip systems, we hypothesize that Case 2 events may result from cross-slip between these systems.

Time-resolved spatial mapping revealed three key features: (i) plastic events formed localized clusters, with Case 1 events primarily located at the core of the clusters and Case 2 events more frequently appearing at the outer boundaries, (ii) events tended to initiate near sample surfaces and migrate inwards with increasing load, and (iii) events in one grain often triggered plasticity in neighboring grains, suggesting intergranular propagation. These findings open avenues for future studies to explore the initiation and propagation of plasticity among grains.

## Supplementary Material

Visualization of grain-scale plastic deformation events. DOI: 10.1107/S1600576725007009/te5153sup1.mp4

## Figures and Tables

**Table 1:** Stiffness tensor components (GPa) for Ti–7Al at room temperature (Lim *et al.*, 2021 ▸)

*C*_11_	*C*_12_	*C*_13_	*C*_33_	*C*_44_
162.4	92	69	180.7	46.7

**Table 2:** CRSS (MPa) for basal, prismatic and pyramidal 〈*a*〉 slip systems of Ti–7Al at room temperature (Pagan *et al.*, 2018 ▸)

Basal	Prismatic	Pyramidal 〈*a*〉
253	248	255

**Table 3:** Total number of grain-scale plastic deformation events detected by Method 1 or Method 2 and validated by Method 3 and/or Method 4

Detection method	No. of events	Method 3	Method 4	Method 3 or 4	Method 3 and 4
Method 1	208	71%	69%	80%	60%
Method 2	288	65%	62%	77%	50%
Method 1 and 2	137	82%	85%	91%	77%

**Table 4:** Total number of Case 1 and Case 2 grain-scale plastic deformation events detected by Method 1 or Method 2 and validated by Method 3 and/or Method 4

Detection method	No. of events	Method 3	Method 4	Method 3 or 4	Method 3 and 4
Method 1, Case 1	128	77%	70%	81%	65%
Method 1, Case 2	80	61%	69%	79%	51%
Method 2, Case 1	98	81%	71%	86%	66%
Method 2, Case 2	190	56%	57%	72%	42%

**Table:** 

	Case 1			Case 2		
Method 1	Basal	Prismatic	Pyramidal 〈*a*〉	Basal	Prismatic	Pyramidal 〈*a*〉
Validated	30	43	31	12	17	34
Unvalidated	5	11	8	6	2	9

**Table:** 

Method 2	Basal	Prismatic	Pyramidal 〈*a*〉	Basal	Prismatic	Pyramidal 〈*a*〉
Validated	24	39	21	42	39	56
Unvalidated	8	3	3	23	21	9

## Data Availability

The data used in this study are available from the authors upon reasonable request.

## Appendix A

### A1. Calibration and indexing

The calibration process consisted of two steps. First, a CeO_2_ powder calibration was performed to determine the sample-to-detector distance and general detector geometry, and second, a ruby calibration was conducted to refine the detector tilt angles and geometry. Both calibrations were carried out at the beginning of the experiment, prior to the initial loading step. The calibration quality was confirmed by full completeness and relatively low χ^2^ values in the ruby indexing results.

After calibration, the sample remained fixed on the sample stage throughout the entire creep loading experiment. Therefore, no additional calibrations were performed during the experiment and fixed detector parameters were used for all subsequent scans.

For grain indexing, a consistent initial orientation list was applied across all scans. Orientation, position and strain tensor parameters were then refined independently for each scan during the indexing process. Thus, static grain tracking was used, with dynamic refinement of grain properties at each scan step.

### A2. Relative volume calculation

The relative volume of the grains was estimated from the integrated intensity of their diffraction peaks. Specifically, the relative volume of each grain was calculated by summing the integrated intensity of its {100} diffraction peaks and normalizing it by the total integrated intensity of all {100} peaks across grains at the initial loading step (unload). To account for any missing {100} peaks, the intensity for each grain was also normalized by the number of detected peaks (Roumina *et al.*, 2022 ▸).

### A3. Diffraction peak extraction

The procedure for isolating each diffraction peak from the raw data and removing background noise is as follows:

(i) The indexed diffraction peaks from HEXRD are first output with their center coordinates (*x*_c_, *y*_c_, ω_c_). A 3D region centered at each peak is then cropped with a fixed window size (*x*_range_, *y*_range_, ω_range_), which is chosen to be sufficiently large to encompass the entire diffraction peak in 3D space. Occasionally, background noise or neighboring diffraction peaks may also fall within the cropping window, and these are subsequently filtered out in the following steps.

(ii) Within each cropped volume, we analyze individual 2D detector images at each ω step. For each frame, we identify clusters of illuminated pixels on the basis of intensity clustering. The cluster containing the peak center is treated as the reference.

(iii) Other clusters are then compared with this reference. To be considered part of the same diffraction peak, a cluster must be spatially connected to neighboring frames (*i.e.* no missing ω slices) and show consistent center positions in the *XY* plane, ensuring overlap or proximity to (*x*_c_, *y*_c_).

(iv) Clusters that do not satisfy these criteria, *i.e.* those that are spatially isolated or discontinuous in ω, are considered background noise or neighboring diffraction peaks and are masked by setting their intensity values to zero.

This approach helps preserve the shape and continuity of the diffraction peak while effectively filtering out unrelated noise or neighboring diffraction peaks that fall within the cropping window.
